# Molecular Marker-Assisted Mapping, Candidate Gene Identification, and Breeding in Melon (*Cucumis melo* L.): A Review

**DOI:** 10.3390/ijms242015490

**Published:** 2023-10-23

**Authors:** Durre Shahwar, Zeba Khan, Younghoon Park

**Affiliations:** 1 Department of Horticultural Bioscience, Pusan National University, Miryang 50463, Republic of Korea; dsh92@pusan.ac.kr; 2 Center for Agricultural Education, Faculty of Agricultural Sciences, Aligarh Muslim University, Aligarh 202002, India; zkhan.agr@amu.ac.in; 3 Life and Industry Convergence Research Institute, Pusan National University, Miryang 50463, Republic of Korea

**Keywords:** quantitative trait loci, genetic and molecular analyses, melon, *Cucumis melo* L., marker-assisted selection

## Abstract

Melon (*Cucumis melo* L.) is an important crop that is cultivated worldwide for its fleshy fruit. Understanding the genetic basis of a plant’s qualitative and quantitative traits is essential for developing consumer-favored varieties. This review presents genetic and molecular advances related to qualitative and quantitative phenotypic traits and biochemical compounds in melons. This information guides trait incorporation and the production of novel varieties with desirable horticultural and economic characteristics and yield performance. This review summarizes the quantitative trait loci, candidate genes, and development of molecular markers related to plant architecture, branching patterns, floral attributes (sex expression and male sterility), fruit attributes (shape, rind and flesh color, yield, biochemical compounds, sugar content, and netting), and seed attributes (seed coat color and size). The findings discussed in this review will enhance demand-driven breeding to produce cultivars that benefit consumers and melon breeders.

## 1. Introduction

Melon (*Cucumis melo* L.; 2n = 2x = 24) is an important diploid, cucurbit vegetable crop grown on approximately 1.5 million hectares worldwide. The total production of melons exceeds 30 million metric tons on a commercial scale in over 100 countries [1]. The global market for vegetable seeds, including melon, was valued at $8.7 billion in 2020 and is expected to reach $15.3 billion by 2027 [2]. Melon, together with watermelon (*Citrullus lanatus* [Thunb.] Matsun. & Nakai), cucumber (*Cucumis sativus* L.), and squash (*Cucurbita* spp.), belongs to the Cucurbitaceae family, which consists of approximately 90 genera and 750 species [3]. It is the most extensively cultivated crop in warmer (tropical and subtropical) regions worldwide. The *Cucumis* genus is divided into two subgenera, *Cucumis* and *Melo*. Melon has been categorized into two subspecies, *C. melo* ssp. *melo* and *C. melo* ssp. *agrestis*, based on differences in ovary pubescence [4].

Melons were domesticated approximately four thousand years ago [5]. Because of the long tradition of melon cultivation and the prevalence of wild, inedible varieties, some experts suggest that India is the center of melon domestication. However, historical records and archaeological evidence indicate that melon was initially cultivated in Egypt and Iran, and later spread throughout the Middle East and Asia, gaining importance as a vegetable in countries such as India, Egypt, Iran, and China [6]. The nearest relative of the melon is *Cucumis picrocarpus* F. Muell., which grows in the wild in Australia [7]. Melon domestication has led to the development of a diverse array of cultivars with different fruit shapes, sizes, colors, and flavors. The Cantalupensis group includes the most diverse melon varieties, which are divided into several subgroups based on geographic origin, fruit morphology, and flavor.

Melon plants are herbaceous, tendril-bearing annuals with fibrous roots and vivid yellow flowers that spontaneously self- and cross-pollinate. Cultivated melons, similar to many contemporary cucurbit plants, were initially monoecious; gynoecious and andromonoecious cultivars developed later. The fruits have minute tubercles and spines of trichome origin on the rind, and range in shape from spherical to ellipsoid or narrowly cylindrical. Melons are usually consumed fresh as snacks or desserts, as such or in fruit cocktails, smoothies, salads, or as ingredients in savory dishes. The fruit is a source of many essential nutrients (Ca, Mg, P, K, Fe, and Zn); sugars (glucose, sucrose, and fructose); organic acids (myristic and pantothenic acid); amino acids; phytochemical compounds, such as carotenoids (α- and β-carotene); vitamins (A, C, E, thiamin, riboflavin, and niacin); fiber; and antioxidant enzymes. Two significant factors that affect fruit quality are the aroma profile and amount of carotenoids in the flesh. Carotenoids function as photoprotectors, visual attractants, and the precursors of fragrance compounds and phytohormones; carotene is a highly abundant carotenoid and is a primary source of vitamin A in food [8,9].

Melons exhibit the highest genetic diversity of phenotypic and biochemical traits, depending on the climatic zone and local preferences of the Cucurbitaceae family, allowing plant breeders to develop superior cultivars [10]. The vital characteristics of the product profiles in melon varieties include desirable plant architecture traits including plant height (dwarfism and branching), flower traits (sex expression and male sterility), fruit quality (sugar and volatile and aromatic compounds), external fruit characteristics (fruit rind color, fruit shape, flesh color, rind netting, and fruit yield), and seed attributes (seed coat color and size). Plant architecture has a large impact on crop productivity, crop quality, and cultivation management. It is a key component in light reception, photosynthate production, and nutrient partitioning in plants. Growth habits (plant height or branching) and flowering characteristics (including sex expression or male sterility) are important breeding traits related to plant architecture.

Fruit traits are crucial attributes that affect consumer preferences and the concomitant selection and breeding of market-preferred varieties. Melon fruit yield is an important trait that differs substantially among cultivars, influenced by genetics, environmental factors, and cultivation practices. Melon fruit yields have been documented to vary between 6101 and 25,173 kg per hectare. In a study of 85 diverse melon varieties, the average yield per plant was found to range from 2.47 to 6.76 kg [11]. Melon seeds are used as food ingredients because they are excellent sources of functional and nutritional compounds. They are rich in beneficial compounds like tocopherols, phospholipids, and sterols, which promote human health. Factors such as seed coat pigment variations impact seed quality; seed coat color can influence biochemical attributes. Breeders often prioritize seed size as a key trait. Research indicates that the seed coat plays a role in water absorption and the determination of seed dormancy. Melon fruits are rich in specific biochemical compounds, such as sugars, carotenoids (particularly beta-carotene), flavonoids, polyphenols, and phytochemicals. The sugar content, mainly composed of sucrose, glucose, and fructose, is a primary target for melon crop development. In addition to the sugar content, the volatile aromatic components of the fruit play integral roles in determining the sensory quality.

The advent of numerous molecular markers has enabled genetic analyses and molecular marker-assisted breeding (MAB) for qualitative and quantitative traits. Numerous commercially important traits of melons, including the yield and fruit quality, exhibit polygenic inheritance. Identifying quantitative trait loci (QTLs) using traditional breeding methods is challenging because of their complexity and interactions with the environment. In marker-assisted selection (MAS), a molecular marker associated with a particular characteristic is indirectly selected to supplement conventional breeding. Thus, genes that influence desirable characteristics can be selected using MAS. When used in conjunction with conventional selection methods, MAS has been confirmed to be an effective tool for selecting plants with desirable traits. Several genomic regions and candidate genes associated with essential horticultural traits, such as fruit quality attributes, ripening behavior, and carotene content, have been identified using QTL mapping analysis in molecular breeding programs [12,13,14,15,16]. Genetic linkage maps and molecular markers for the QTLs of horticultural traits in melons identified in previous studies are briefly reported in Table 1. Furthermore, the identification of large numbers of single nucleotide polymorphisms (SNPs) using whole-genome resequencing (WGRS) has facilitated the rapid identification and definition of candidate genomic areas for fruit quality attributes in melons [17]. Comparative genomic analysis has also been used to identify functional genes that govern both the qualitative and quantitative phenotypic traits of melons [18].

Genetic map construction is a reliable and crucial step in QTL detection and gene mapping. A limited number of markers, such as cleaved amplified polymorphic sequences (CAPS), simple sequence repeats (SSRs), restriction fragment length polymorphisms (RFLPs), amplified fragment length polymorphisms (AFLPs), and random amplified polymorphic DNA (RAPD), have been exploited for generating genetic linkage maps for QTLs for various phenotypes in several populations [23,25,26,27]. These maps were primarily used to identify QTLs for agronomic traits. A high-resolution genetic map using 580 SNPs, anchoring 354.8 Mb sequences, was used to improve the melon genome considerably [28], which was published in 2012 [29], and developed from a double-haploid line created by breeding two melon cultivars, *C. melo* and *C. agrestis*. Numerous research projects have successfully located and created markers for traits such as plant architecture, floral biology, fruit and seed quality traits, and biochemical compounds [30].

Markers created for MAS applications must be closely related to genes or QTLs. It has been widely acknowledged that MAS must consider QTL validation, QTL effects, or fine-mapping with high resolution [31]. Molecular markers regulate gene expression and have been associated with candidate genes for particular traits, with extensive applications in parental selection, genetic diversity analysis, and targeted MAS. Numerous technologies have been developed including linkage mapping, QTL mapping, genotyping-by-sequencing (GBS), WGRS, genome-wide association studies (GWAS), bulked segregant analysis (BSA), QTL sequencing (QTL-seq), and RNA sequencing (RNA-seq), in which molecular markers have been utilized for the identification of QTLs and functional genes for particular traits. The availability of a reference genome sequenced in 2012 has enabled the implementation of this technology in melons. The melon genome is relatively small, comprising 375 Mbp and 27,427 protein-coding genes [29]. The melon genome sequence and available germplasm resources have aided in the discovery of genetic modifications and provided genome-wide insights into melon domestication [29]. In addition to MAB, genetic engineering is a widely used technology that contributes substantially to genetic advancement and varietal evolution by reducing the risk and restrictions of conventional breeding methods. This approach is effective in other crops but is not yet common in melons.

This review outlines the genetic regulation of qualitative and quantitative traits in melons to assist trait selection and integration, as well as the development of novel varieties with enhanced yield and other desirable horticultural characteristics. This review discusses the QTLs, major and minor functional genes, and the development of molecular markers related to horticultural traits, including plant architecture, branching patterns, floral biology, fruit and seed traits, and biochemical compounds.

## 2. Molecular Markers, QTLs, and Candidate Genes for Horticultural Traits in Melon

### 2.1. Plant Architecture

#### 2.1.1. Dwarfism

Dwarf and bush melon varieties are commercially important because of their concentrated fruit sets, lodging resistance, early maturity, tillering ability, and potential for planting at higher densities than regular vining types. Several dwarf crop traits, including short internode (SI) length, short mainstem length, and bush-type growth habits, have been reported in cucurbits. Plant dwarfism is primarily caused by mutations in the genes associated with hormonal biosynthetic pathways, including those that produce gibberellins (Gas) [32], cytokinins [33], and brassinosteroids (BRs) [34]. Dwarfism in melons is also controlled by hormonal pathways, and a few genes have been identified as candidates for regulating dwarfism in melons. Hwang et al. [35] generated dwarf and vine plant types by crossing the melon mutant line, PNU-D1 (*C. melo* ssp. Cantalupensis), with the inbred, wild-type melon line, PNU-WT1 (*C. melo* ssp. Agrestis). In their study, the primary stem length of the F_2_ progenies indicated that a single recessive gene, *mdw1*, controlled dwarfism in this population. A genetic linkage map was constructed using SSR markers to determine the chromosomal location of *mdw 1* and 76 SSR markers positioned on 15 linkage groups (LGs) spanning 462.84 cM localized the *mdw1* to Chr. 7. They identified the candidate genes, *ERECTA (serine*/*threonine kinase)* and *UBI (ubiquitin)*, in the genomic regions flanking *mdw1* at distances of 0.6 and 1.2 cM, respectively.

In watermelons, Cho et al. [36] constructed a genetic map of F_2_ and F_2:3_ populations derived from a cross between a “Bush Sugar Baby” (BSB, semi-dwarf type) and a PCL-J1 (normal type) cultivar and identified the QTL for semi-dwarfism (*sdw*) on Chr. 9 in watermelons using GBS. Furthermore, they performed QTL sequencing and identified a genomic region that matched the *sdw-1* flanking region in the linkage map. After narrowing down the genomic region, they identified a candidate gene, *ClCG09G018320*, encoding the *ATP-binding cassette (ABC) transporter B family member 19*, which controls semi-dwarfism in BSB.

In cucumber, genome-wide molecular mapping with SSR markers was conducted using two inbred cucumber lines, PI 308915 (compact vining) and PI 249561 (regular vining), and a linkage map consisting of 187 SSR markers covering 537.5 cM was identified. Linkage analysis placed the *cp* locus at Chr. 4. After narrowing the *cp* locus to a genomic DNA region spanning 220 kb, an annotated gene with a predicted function was identified. This gene is a homolog of the *cytokinin oxidase (CKX*) gene and can serve as a candidate compact gene [37]. Zhang et al. [38] generated a set of 186 F_2_ plants derived from a cross between the pumpkin inbred lines, “Rimu” and SQ026. The F_2_ plants were genotyped using a GBS approach. A high-density genetic map containing 458 binary markers was constructed. Three QTLs in LGs 1, 3, and 4 were identified. One QTL, *qCmB2*, which was located at an interval of 0.42 Mb on LG 3, explained 21.4% of the phenotypic variations and a candidate gene, *Cma_004516*, encoding *gibberellin (GA) 20-oxidase* in the GA biosynthesis pathway was identified, suggesting that this gene is a possible candidate gene controlling the dwarf vine in pumpkin [38].

#### 2.1.2. Branching

Melons are monoecious or andromonoecious, with male flowers located on the main stem and female or hermaphroditic flowers on lateral branches; the plant grows on a long, trailing vine. Therefore, lateral branching is a key element in melon plant architecture. Three main growth patterns are evident in muskmelons: vining, SIs, and bird nests (multiple branches). Fruit is often set on vining types away from the center of the plant. As the fruits do not mature consistently, they are harvested separately. Cultivating and controlling weeds is challenging as the vines extend between rows. SI muskmelons have fewer and shorter internodes and more restricted leaf surfaces per plant than vining muskmelons [39]. According to Nerson et al. [40] and Paris et al. [41,42], bird nest melons are unique because of their compact growth habits and the capacity to germinate quickly at low temperatures. Fruits are placed close to the center of the plant, resulting in uniform growth and maturity [41]. In addition, these melons have a comparatively concentrated fruit-setting time and a homogeneous, highly branched plant habit with shorter internodes [42]. Therefore, high yields, high-quality fruits, and cost savings in crop management (including various environmental and endogenous signals) can be achieved by maintaining the lateral branches in good condition [43].

Fukino et al. [44] used 94 F_2_ plants resulting from a hybrid between “Chukanbohon Nou 4 Go” (Nou-4) and “Earl’s Favorite” (Harukei-3) to create a linkage map to uncover molecular markers for MAS of the *short lateral-branching* (*Slb*) gene. Nou-4 is a weedy melon with a short, lateral-branching trait. This characteristic is governed by a single recessive or partially dominant major gene called *slb*. QTL analysis of the F_2_ population revealed two loci for short, lateral branching. With an LOD score of 12.5, a major QTL in LG XI, in which the *slb* allele from Nou-4 has been associated with short, lateral branching, explained 50.9% of the phenotypic variance. With an LOD score of 4.2, another minor QTL in LG III, where the *slb* allele from Harukei-3 explained 9.9% of the phenotypic variance, was related to short, lateral branching. This study also showed that a QTL in LG XI (*slb*) coupled to an SSR marker could be utilized to select short, lateral branching.

Fang et al. [43] identified four homologs of *SHORT VEGETATIVE PHASE (SVP)-*like genes, including *CmSVPa*, *CmSVPb*, *CmSVPc*, and *CmSVPd*, in melons; the gene structure of *CmSVPc* differed from those of the other *CmSVP*-like genes. Overexpression of *CmSVPc* in *Arabidopsis* leads to increased lateral branches. However, *CmSVPc-*RNAi plants in melons demonstrated the repressed formation of lateral branches, shorter internodes, reduced petal size, and more compact fruits compared to the control plants, suggesting that *CmSVPc* might be a candidate gene for maintaining the growth of lateral branches in melons. A lateral branchless trait was also identified in watermelons from the inbred WCZ, and genetic analysis revealed that it was controlled by a single recessive gene, *Clbl* (*Citrullus lanatus branchless*) [45]. Linkage analysis and BSA-seq were used to map *Clbl* in the watermelon Chr. 4. This demonstrated that the candidate gene of branchless watermelons, *Cla018392*, belongs to the *TERMINAL FLOWER 1* (*TFL1*) gene family, implying a genetic control for lateral branching that is different from that in melons.

### 2.2. Flower Traits

#### 2.2.1. Sex Expression

The floral biology and sexual expression of melon flowers are crucial characteristics in melon improvement programs. Commercial melon cultivars are typically andromonoecious and produce male and bisexual (perfect) blooms in the developmental stages. To avoid artificial emasculation and ensure hybrid purity, gynoecious sexual expression, which produces female or pistillate flowers, is extremely valuable in melon heterosis breeding. In melons, two key genes have been implicated in regulating sex expression: *andromonoecious* (*a*) and *gynoecious* (*g*), or *CmACS-7* and *CmWIP1*, respectively. The allele “*a*” regulates the presence or absence of the pistil in female flowers, whereas the allele “*g*” regulates the presence or absence of two different types of flowers on the same plant [46]. Four distinct genotypes are produced by different allelic combinations at these two loci: hermaphrodite (*aagg*), gynoecious (*Aagg*), monoecious (*A_ G_*), and andromonoecious (*aaG_*) [47]. Locus *a* encodes the enzyme, *1-aminocyclopropane-1-carboxylic acid (ACC) synthase (ACS*), which prevents the development of stamens in female flowers [48]. The primary stage in the synthesis of ethylene is the production of *ACS*, which is encoded by the *CmACS-7* on Chr. 2 and is homologous to the *ACS-7* gene in *Arabidopsis* [48].

Kim et al. [49] developed molecular markers for monoecious plant selection based on the sequence variation inherent in *CmACS-7*, which is required for ethylene synthesis. Complete sequences of *CmACS-7* were cloned from monoecious (MO23) and andromonoecious (AM24) lines, and the alignment of these *CmACS-7* sequences revealed an SNP (C170T) in exon 1 and an 18 bp indel in the 3′-untranslated region (UTR) between MO23 and AM24. Subsequently, the SNP and indel were used to develop a CAPS (*EX1-C170T*) and a sequence-characterized amplified region (SCAR) marker (*T1ex*). Sex expression was determined in 442 F_2_ plants obtained from a cross between the MO23 and AM24 plants. The sex types of 429 plants (13 not classified) co-segregated with the SCAR marker, indicating that sex expression mediated by *CmACS-7* is controlled by a single dominant gene that confers monoecy in the line, MO23. Daryono and Prasetya [50] investigated the expression of *CmACS-7* in different sexual forms of melon flowers using RT-PCR and found a higher expression in female and hermaphrodite flowers than that in male flowers. Moreover, the expression of *CmACS-7* was higher in male flowers with an andromonoecious sexual type than in male flowers with a monoecious sexual type. Recently, a GWAS revealed the SNPs on Chr. 1 and 8 associated with sex expression in the oriental melon and identified four promising candidate genes: *MELO3C015898* (*transport inhibitor response 1*), *MELO3C015904* (*SWR1-complex protein 4/DNA methyltransferase 1-associated protein 1*), *MELO3C024563* (*putative UDP-N-acetylglucosamine—peptide N-acetylglucosaminyltransferase SPINDLY*), and *MELO3C024565* (*MRNA-decapping enzyme-like protein*) [51].

Sex expression in developing flower buds is plastic; therefore, the sex of any particular flower at a given node depends on various genetic, developmental, environmental, and hormonal factors. External variables, such as mineral nutrition, temperature, water regulation, illuminance, photoperiod, mechanical trauma, and the use of growth regulators, can alter sex expression in melons [52]. Growth regulators or phytohormones, particularly ethylene/ethrel [52,53] and gibberellic acid, GA3 [54], have mostly been employed to modulate melon sex expression. Ethylene production in melons corresponds to the development of the pistil in flowers, whereas ethylene biosynthesis inhibitors suppress the growth of pistillate flowers and promote that of staminate flowers [50]. In brief, the regulation of floral biology and sexual expression in melons is a complex process involving key genes such as *CmACS-7*. Additionally, environmental factors and growth regulators can influence sexual expression, highlighting the plasticity of this trait in melons.

#### 2.2.2. Male Sterility

Male sterility (MS) is a key feature observed in various higher plants and is essential for producing low-cost hybrid seeds because it eliminates the requirement for cross-pollination and emasculation. Disorders in tapetum formation and abnormal pollen growth are closely related to the occurrence of MS. The abortion of pollen results from mutations in the genes at various pollen developmental stages such as *Dysfunctional Tapetum 1* (*DYT1*) [55], *ABORTED MICROSPORES* (*AMS*) [56], and *basic Helix–Loop–Helix* (*bHLH*) [57] transcription factors identified in melon anthers.

Genetic male sterility (GMS) in melons involves five recessive genes, *ms-1* to *ms-5*. ”Punjab anmol” [58], and “MH-27” [59], which are commercial F_1_ hybrids, were successfully developed using the *ms-1* GMS inbred line. To introduce MS into fertile melon cultivars and breeding lines to construct parents for F_1_ hybrid seed production, Park and Crosby [60] developed a SCAR marker (*SOAM08.644*) that was tightly linked to the *ms-3* gene at 2.1 cM on the LGs. A linkage map was constructed based on SSR markers for the *ms-1* locus using an F_2_ population from a cross between MS-1 and KP4HM-15 [61]. Linkage analysis of the F_2_ plants mapped three SSR markers, *DM0187*, *DM0038*, and *TJ14*, to *ms-1* on Chr 6. Of these, marker *DM0187* was closest to the gene at a genetic distance of 4.8 cM and was successfully used to transfer the *ms-1* (male sterility) gene into elite muskmelon genotypes through MABC [61]. Subsequently, the candidate gene for *ms-5* was mapped to a 30 kb genomic region on Chr. 9 using BSA combined with specific length amplified fragment sequencing (SLAF-seq) [62]. The CAPS markers which were developed from this location based on parental line resequencing data were confirmed by the mapping of 252 F_2_ individuals.

Furthermore, six genes were annotated in the 30 kb genomic region, of which the *LOC103498166 AMS* gene was expressed differentially during the pollen tetrad formation period in MS lines (ms-5) and male-fertile (HM1-1) lines, suggesting that *AMS* is a possible candidate gene for MS in melon [62].

### 2.3. Fruit Traits

#### 2.3.1. Fruit Rind Color

The rind color in melons varies from yellow-green, light to dark green, and orange. Melons can also have mottled or striped rinds. The dark- and light-green rinds of melon fruit exhibit qualitative variations in the chlorophyll content. Prior research has found that in a biparentally segregated population, a light immature rind color exhibits recessive single-gene inheritance [63]. Green rind color was dominant and epistatic to non-green (white and yellow) colors when analyzing an F_2_ segregation population from a cross between green and yellow rind accessions [64]. Oren et al. [65] performed a GBS-based GWAS using two segregating biparental populations. The initial population consisted of 164 recombinant inbred lines (RILs) at the F_7_ generation, resulting from a cross between a light rind, honeydew parent (“Tam Dew”), and a dark rind, reticulatus parent (“Dulce”). The second population comprised 114 families at the F_3:4_ generations, obtained by crossing “Dulce” with another light rind accession of yellow casaba, inodorous melon. The GWAS identified a genomic region (640–930 kb) on Chr. 4 that is associated with the rind color. Furthermore, they mapped the target genomic region and found 33 putative genes, including *MELO3C003375* annotated as an *ARABIDOPSIS PSEUDO-RESPONSE REGULATOR2-LIKE* gene (*APRR2*), that were responsible for the light rind phenotype in melons [65]. The aforementioned gene is the melon ortholog of a recently identified causal gene in cucumbers with a recessive white rind (*w*) mutation [66].

Gur et al. [67] characterized yellow or white/cream rind color traits in multiple melon accessions using GWAS and found that two SNPs at positions 3,541,676 and 3,541,866 bp on Chr. 10 exhibited the strongest association with yellow rind color and were placed approximately 65 kb upstream and eight genes away from the previously identified *CmKFB* (*MELO3C011980*). Yellow and white rind accessions were genotyped using a PCR marker at *CmKFB* to validate SNP3541676 and SNP3541866; co-segregation was demonstrated using the QTL marker for yellow and white rind color.

Later, Zhao et al. [64] detected candidate genes associated with rind color (green), *MELO3C003375* on Chr. 4 encoding the *two-component response regulator-like protein*, *APRR2*, and the previously identified gene, *CmKFB* (*MELO3C011980*), on Chr. 10, which negatively regulated naringenin chalcone accumulation. Naringenin chalcone is a yellow flavonoid pigment that is considered one of the primary pigments affecting melon rind color. *MELO3C003375* was expressed at much higher levels during the gene expression test than in the white and yellow rind-colored lines. However, *CmKFB* was hardly expressed in yellow rind color lines, which was consistent with its function of negatively regulating flavonoid accumulation, suggesting that *MELO3C003375* and *CmKFB* are associated with green and yellow rind colors, respectively. An additional gene, *MELO3C003097*, was detected by Zhao et al. [64] on Chr. 8. This gene is an ortholog of *Arabidopsis SG1*, which encodes the protein *SLOW GREEN 1* and is required for chloroplast development, suggesting that *MELO3C003097* might be a minor gene involved in the formation of green, white, and yellow rind colors.

Shen et al. [15] reported that two dominant epistatic genes, *CmMt1* and *CmMt2*, regulate the formation of mottled rinds by mapping the F_2_ population derived from SC (mottled rind) and MG (non-mottled rind). The observed phenotypic segregation ratio suggested that immature rind color exerted an epistatic influence on the mottled rind phenotype. This effect was regulated by a specific gene, *CmAPRR2*. To further validate this, a Kompetitive allele-specific PCR (KASP) DNA marker, *CmAPRR2SNP(G/T*), was developed, which demonstrated co-segregation with the rind color trait, suggesting that *CmAPRR2* corresponded to *CmMt1*. Using BSA-seq and KASP markers, *CmMt2* was successfully fine-mapped to a specific region of 40.6 kb, containing six predicted genes. *MELO3C026282*, a homolog of *AtCPSFL1*, emerged as the top candidate gene for *CmMt2* based on functional annotation, expression analysis, and sequence variation analysis. Additionally, investigations involving pigment content measurements and transmission electron microscopy have revealed that *CmMt2* is likely to play a role in chloroplast development, subsequently leading to reduced chlorophyll accumulation [15]. Shen et al. [15] attributed the mottled rind phenotype in melons to abnormal chloroplast development and altered chlorophyll accumulation during fruit development. It was also proposed that changes in the developmental stage of the thylakoid structure within the chloroplasts may account for the disparity in immature rind color between the two parental lines. Numerous genes affect rind color, and the genetic mechanism underlying this trait is intricate. Understanding the genetic basis of these traits is crucial for targeted breeding efforts to develop market-preferred varieties with desirable fruit rind color in melons. These genes must be functionally verified before they can be used for breeding. Genes controlling other rind colors in melons remain unknown.

#### 2.3.2. Fruit Shape

Melon fruit can be round, elongated, or oblong. Fruit shape was determined using the fruit shape index (FSI) and the ratio of fruit length (FL) to fruit diameter (FD). Various QTLs for FSI have been identified and are related to QTLs for FL and FD [19,22,27,68,69]. Using an F_2_ population from the cross “Piel de Sapo” × PI124112, Diaz et al. [12] identified ten QTLs, five QTLs for FL on Chr. 2, 3, 6, 8, and 10, two for FD on Chr. 3 and 12, and three for fruit shape on Chr. 2, 8 and 12. Gur et al. [67] found SNPs in a previously identified QTL region for fruit shape on four Chr. (2, 8, 11, and 12) and mapped the fruit shape using GWA analysis on 177 inbred accessions of 2 melon subspecies (spp. *C. agrestis* and *melo*). Pereira et al. [70] identified 17 QTLs associated with FL, FD, FSI, fruit weight (FW), and fruit perimeter (FP). Utilizing a high-density genetic map precisely localized the major-effect QTLs, *FW5.1*, *FD5.1*, and *FP5.1*, within a 496 kb genomic region containing 48 predicted genes. Among these genes, *MELO3C014402*, which encodes the protein FANTASTIC FOUR 2, was suggested to be a potential QTL for FW [70]. Later, Ma et al. [71] used a gene expression test and RT-PCR to identify 24 *CmSUN* family genes and found that the expression of most *CmSUNs* was specifically enriched in the reproductive organs of melons, including young flowers and ovaries. Furthermore, the overexpression of *CmSUN23-24* and *CmSUN25-26-27c* resulted in increased FSI, indicating that these genes are important regulators of melon fruit shape variation. Using a BSA-seq from B8 (long-horn fruit) and HP22 (flat-round fruit) melon inbreds, Ma et al. [16] identified a genomic region with a 53.7 kb interval on Chr. 8 for fruit shape. They narrowed the genomic region and suggested that the fruit shape was controlled by a single dominant locus, *CmFSI8/CmOFP13*, which encodes the OVATE family protein (OFP) (Table 2). In addition, they developed allele-specific markers for *CmFSI8* and validated these markers in a segregating population.

#### 2.3.3. Fruit Flesh Color

The color of melon fruit flesh is determined by chlorophyll and carotenoid pigments, resulting in distinctive white, green, and orange colors [76]. β-carotene is the major carotenoid found in orange-fleshed melon varieties [77], and most variations in the color intensity are caused by quantitative variations in the β-carotene content [76]. Numerous studies have identified several QTLs that govern this trait in various genetically distinct species [19,20,21,22,23,24,25,26,27,28,29,78].

Fruit flesh color in melon is a complex trait that is quantitatively controlled by two major epistatic genes: green flesh (*gf*) and white flesh (*wf*). The presence of *Gf* determines the orange flesh, which is dominant over the green flesh (*gf*). Melons with *gfgf* have either white flesh (*wfwf*) or green flesh (*Wf-*) [79,80]. The gene *CmOr*, formerly known as the *gf* (green flesh) locus in melons, was found to co-segregate with fruit flesh color in segregating F_2_ and backcrossing populations derived from a cross between light-green (“Tam Dew”) and orange (“Dulce”) flesh melon genotypes and presented two haplotypes (alleles) of the *CmOr*-encoding *protein ORANGE-ORANGE*, *chloroplastic*, one of which was associated with orange flesh and the other with either white or green flesh [81]. A specific “golden SNP” was identified in *CmOr* and was pivotal in determining the orange and non-orange phenotypes observed in melon fruit [81]. Overexpression of the orange allele of *CmOr* in the *Arabidopsis* callus system stimulated the accumulation of β-carotene. Through site-directed mutagenesis of the *CmOr* green/white allele, the substitution of arginine with histidine resulted in a marked increase in β-carotene accumulation.

Gur et al. [67] characterized the flesh color variation using GWAS. They analyzed flesh color in longitudinal sections of 177 inbred accessions derived from 2 melon subspecies (ssp. *C. agrestis* and ssp. *melo*) and found that color phenotypes were uniformly distributed within the GWAS panel, with green, white, and orange color categories representing substantial proportions. Using GWAS, they identified a single SNP on Chr. 9 (at position 20,550,439 bp) that had a substantial effect; this SNP was located within the causative *CmOr* gene (*MELO3C005449*), indicating that ~70% of the flesh color variation was across the panel and effectively distinguished between orange and non-orange. Additionally, all lines were genotyped for polymorphisms in *CmOr* to validate SNP20550439, exhibiting a complete co-segregation of *CmOr* with orange and green flesh colors (Table 2).

Galpaz et al. [73] conducted a QTL analysis of the flesh color in melons using an RIL population and identified two major loci, including the *CmPPR1* gene (*MELO3C003069*) on Chr. 8 as a candidate for white flesh. *CmPPR1*, which encodes a member of the *pentatricopeptide protein* family, is involved in RNA processing in plastids, where carotenoids and chlorophyll pigments accumulate. The *CmPPR1* gene is positioned within the 2–5 Mb region previously associated with the *wf* locus in white flesh [81]. Zhao et al. [64] conducted a GWAS on 688 melon accessions and identified a genomic region on Chr. 8 associated with the flesh color, in addition to the previously recognized *Gf* gene (*CmOr*) controlling orange flesh [81]. Notably, this genomic region overlaps with the *Wf* locus, which regulates white and green flesh, as reported by Perin et al. [82].

To further identify candidate genes, researchers developed an RIL population from a cross between an orange flesh line, “Védrantais,” and a white flesh parental line, “Piel de Sapo,” for QTL mapping. They identified a significant QTL corresponding to the *Wf* locus on Chr. 8. When the QTL mapping and GWAS results were combined by Zhao et al. [64], a 96 kb region carrying 11 protein-coding genes was found to be strongly associated with the QTL. A previously identified candidate gene, *MELO3C003069 (CmPPR1*), by Galpaz et al. [73] for *Wf*, was 202 kb away from the mapping interval found in the study. One of the 11 genes, *MELO3C003097*, which is the ortholog of *SG1* in *Arabidopsis* and has been reported to be essential for chloroplast development and chlorophyll biosynthesis [65], showed a considerably higher expression throughout fruit development in green-fleshed accessions than in white-fleshed accessions, suggesting that *MELO3C003097* is a promising candidate gene for the *Wf* locus in green flesh. CAPS markers have been utilized for the MAS of fruit flesh color, rind color, and shape attributes [67].

#### 2.3.4. Fruit Rind Netting

The physical appearance of fruits of different melon varieties varies depending on the skin surface; fruits of some varieties have a smooth skin appearance, whereas others exhibit different types of reticulation decorations, referred to as netting. Reticulation, or rind netting, is a term for ligno-suberized patterns that vary in degree and beautify the melon cuticle [83]. Netting originates from cracks that appear on the fruit surface. During the time of maximum developmental rate, the rind of the smooth-rind variety has a lower cuticle deposition than netted fruit [84]. Rind characteristics, such as thickness and netting, can be associated with shelf life and resistance to storage and shipping [85].

Few studies have been conducted to identify the QTL associated with rind netting in melons. Based on the QTL analysis in Trial 1, *qND6* on LG6 was the sole QTL identified for net density (ND). Notably, this QTL co-localized with *qNW6*, one of the four QTLs mapped for net width (NW) [26]. In Trial 2, three additional QTLs for ND (*qND2*, *qND4*, and *qND7*) in LG2, LG4, and LG7, respectively, were found to co-localize with QTLs for NW (*qNW2*, *qNW4*, and *qNW7*). These findings suggest a strong correlation between ND and NW, with NW influencing ND. However, only two QTLs associated with NW (*qNW2* and *qNW7*) were consistently detected in both trials [26]. Furthermore, the authors reported new QTLs related to the netting density (*qND4*, *qND6*, and *qND7*) and netting width (*qNW2*, *qNW4*, *qNW6*, and *qNW7*) [26]. Two QTLs for rind ND, *NDen2.1* and *NDen2.2*, were identified on Chr. 2 across 9 and 2 Mb, respectively, with a genetic interval of 12 cM [83]. A third QTL, *NDen9.1*, was reported on Chr. 9, with a genetic interval of 12 cM across 734 kb [83]. All three QTLs (*NDen2.1*, *NDen2.2*, and *NDen9.1*) are responsible for the variation in rind ND [83].

Various QTLs for rind thickness and netting have been identified in the introgression lines of melon var. *makuwa*, of which *Rth.6* for rind thickness was co-localized on Chr. 6 with *net.6*, which accounted for the less netted fruits. Additionally, two other QTLs, *net.5* and *net.7*, were associated with the reduced netting variety in *makuwa* [85]. Wang et al. [26] identified four QTLs related to the NW of the fruit rind that were co-localized with QTLs for ND and proposed that a similar mechanism was responsible for the development of these two traits. Several QTLs associated with ND and NW were identified, indicating a strong correlation between these traits. Liang et al. [86] studied the genetic basis of the skin netting (*CmSN*) locus in melons using GWAS by crossbreeding the H906 line with smooth fruit and the H58 line with netted fruit. They observed that *CmSN* was controlled by a single dominant gene located on Chr. 2 within an area of ~351 kb. Furthermore, they delimited the genomic region to a 71 kb region containing five genes. Among these, *MELO3C010288*, encoding a protein in the EamA-like transporter family, was the most likely gene responsible for the netted phenotype [86].

#### 2.3.5. Fruit Yield and Its Components

Melon fruit yield is an important trait that differs substantially among cultivars. According to FAOSTAT [87], the global production of melons ranges from 27 to 28 million tons. Melon fruit yield is determined by FW, length, and width, which vary considerably among melon cultivars [11,88]. It was reported that melon fruits generally weigh ~0.05–15 kg [89]. Other secondary traits, such as plant height, number of branches, flowers, and fruits per plant, were indirectly associated with fruit yield. Melon fruit yield is also related to various components such as FL, width, and weight. Several QTLs associated with the major components of fruit yield have been discovered and candidate genes for the QTLs have been identified [68,85,90].

Zhang et al. [68] constructed a linkage map with 195 CAPS markers and identified 28 QTLs associated with fruit traits, including FW, length, width, and Brix values, using an F_2_ population derived from the thick-skinned line, “Elizabeth,” (M4-5), and a thin-skinned line, M1-15. Derived-CAPS (dCAPS) markers have been developed for the MAB of fruit-related traits in melons. Most QTLs associated with fruit-related traits (FL, width, and weight) were clustered on Chr. 6 and 9 [68]. The positioning of these QTLs within restricted genomic intervals can facilitate the application of MAS in breeding programs [68]. Amanullah et al. [69] identified QTLs for fruit yield-related traits (FW, length, and width) on Chr. 9 using QTL mapping with SNP-based CAPS markers and WGRS in melons. Later, three QTLs for fruit weight (*FWt2.1*, *FWt4.1*, and *FWt9.1*) and one major QTL for fruit length (*FL12.1*) were mapped, based on SNP-derived CAPS markers in melon [90].

Lian et al. [91] developed genetic maps from two F_2_ populations, WAP and MAP, using WGRS. These populations originated from different crosses involving cultivated and wild agrestis melons. From this study, 871,671 and 1,976,589 high-quality SNPs were identified in WAP and MAP, respectively. They identified two key loci influencing melon fruit size: one on Chr. 11 in the WAP population and another on Chr. 5 in the MAP population. In the WAP population, two QTLs linked to FD and weight were located on Chr. 11, sharing a common 130.8 Kb region. Notably, within this overlap, the *MELO3C025758* gene, encoding an auxin response factor (ARF), was pinpointed as a possible candidate gene in melon fruit size. For the MAP population, a region on Chr. 5 was identified that impacted FW and length. Within this region, the gene, *MELO3C004493*, which encodes a YABBY transcription factor—a type previously linked with tomato fruit size—was found. As a result, both *MELO3C025758* (ARF) and *MELO3C004493* (YABBY transcription factor) are postulated to be candidate genes for melon fruit size [91]

### 2.4. Seed Traits

#### 2.4.1. Seed Coat Color

Melon seeds are rich in vital bioactive compounds, including tocopherols, phospholipids, and sterols, and offer substantial health benefits [92]. Melon seeds possess a variety of colored seed coats, from white, yellowish-white, dark brown, and reddish-brown to other colors, of which white, yellow, and brown are the most typical. An earlier study revealed that the color of the seed coat was related to the amount and activity of antioxidants, as well as the biochemical properties of the seed [93]. Zeb et al. [94] examined the phenolic composition, total phenolic concentration, and antioxidant activity of honeydew melon seeds. Five phenolic compounds, including gallic acid and its derivatives, hydroxybenzoic acid, catechin derivatives, and caffeic acid, were identified in the water extracts using high-performance liquid chromatography with diode array detection (HPLC–DAD). Research indicates that the seed coat plays a role in numerous aspects, including how seeds absorb water [95] and their dormancy patterns [96]. The quality of a seed can also be affected by variations in the color pigments of the seed coat [97] and germination.

To explore the genetic inheritance of seed-related variables using major gene and poly-gene genetic models, F_2:3_ families were produced using cantaloupe inbred ms5 and muskmelon inbred HM1-1 as the parental lines. QTL mapping of the F_2:3_ families showed that a single dominant locus (*SCC*) controlled the seed coat color, and the white color was dominant over yellow [98]. Li et al. [99] used an F_2_ segregation population and SLAF markers and constructed 12 LGs with a map distance of 1356.49 cM (an average genetic distance of 0.37 cM). The locus, *SC12.1*, responsible for the seed coat color (SC) in muskmelon, was located on Chr. 12 within the genomic region, spanning 12,785,226 to 15,029,452 bp. These findings lay the groundwork for future gene cloning and functional analyses to elucidate SC traits in muskmelon [99].

Hu et al. [75] carried out a BSA-seq analysis of an F_2_ population derived from IC2508 with yellow and IC2518 with brown seed coats and identified a genomic region of 11,860,000–15,890,000 bp (4.03 Mb) on Chr. 9. Subsequently, a 233.98 kb region harboring 12 annotated genes was finely mapped and a single dominant gene *CmBS-1* (*MELO3C019554*), encoding *homeobox protein* (*PHD transcription factor*), was identified as a candidate gene for yellow SC. *MELO3C019554* was expressed at lower levels in yellow seed coats than in brown seed coats. It has been reported that *MELO3C019554* is related to 12 flavonoid metabolites.

#### 2.4.2. Seed Size

Seed size plays a crucial role in determining the evolutionary fitness and agronomic characteristics of plants during domestication and breeding. Seed size substantially affects the seed yield, eating quality, and tolerance to environmental stresses [100,101]. The genetic basis of the seed size and weight remains poorly understood; however, recent advances in melon genome sequencing have shown promising results [102,103]. Jiao [104] used an F_2:3_ population resulting from crossing large-seeded inbred ms-5 and small-seeded inbred HM-1 to carry out QTL analysis for seed size. Four chromosomes (Chr. 5, 6, 9, and 11) had seven QTLs for seed length (SL), seed width (SW), and 100 seed weight (100SWT). Two major-effect QTLs, *Sl11.1* and *SW11.1*, controlling SL and SW, were identified in the same genomic region on Chr. 11. They exhibited a marked phenotypic variation explained (PVE) of 17.5% and 19.5%, respectively. Ye et al. [105] identified four QTLs, including two for SL, one for SW, and one for 100SWT, using a BC_1_ population from a cross between MR-1 (long seed) and M1-15 (short/narrow seed). Two QTLs for seed length and seed diameter, *SL5.1* and *SD5.1*, were located on Chr. 5, explaining a PVE of 9.9–11.2%, and two QTLs, SL1.1 and TGW1.2, were positioned on Chr. 1. Later, Pereira et al. [70] identified four QTLs for seed weight using a high-density genetic linkage map of the RIL population derived from crosses between “Védrantais” and “Piel de Sapo,” with one major QTL (*SWQU8.1*) positioned on Chr. 8 with 19.9% PVE.

Zhang et al. [98] mapped six QTLs for SL, SW, and 100SWT in an F_2:3_ population by crossing ms-5 and HM1-1. The major QTLs, *sl11.1* and *sl11. 2*, for SL and *100swt11.1* for 100SWT were identified on Chr. 11, while the QTL, *sd2.1*, for SW was detected on Chr. 2. Furthermore, Amanullah et al. [69] utilized 163 SNP-derived CAPS markers and 148 F_2_ plants derived from M4−75 (female parent with long and thick seeds) and X207 (male parent with small and thin seeds) to map QTLs for SL, SW, seed weight, and seed thickness. They identified three QTLs for SL, SW, and thickness on Chrs 3 and 9. A major QTL, SW3.1, was detected on Chr. 3 with an *R^2^* of 13.32%. Jiao [104] and Zhang et al. [98] detected QTLs for SL, SW, and 100SWT in the same region. This suggests that *Cm11.1* is likely to play a substantial role in regulating the seed size and weight variation in melons. Candidate genes for the seed size in melons have been cloned from the 43.47 kb–1.89 Mb genomic region of the seed trait QTLs (*qSL3* and *qSWD3*) on Chr. 3, using the QTL mapping approach of Zhang et al. [103]. To validate the presence of *qSL3* and *qSWD3* on Chr. 3, they designed and utilized 18 KASP markers within the QTL region. Employing regional QTL mapping analysis, they successfully confirmed the existence of *qSL3* and *qSWD3* within the genomic region delimited by the KASP markers, *A288* and *A291*, spanning approximately 522 kb in length (29,021,264 to 29,543,031 bp) [103]. Furthermore, the mapping region was narrowed down to 522 kb, and 40 genes were annotated, of which *EVM0009818*-encoded *cytokinin-activated signaling* was selected as a candidate gene. Based on RNA-seq data, this gene was differentially expressed in the young fruits of the parent plants. These findings lay the foundation for future research to elucidate the genetics of seed size and ultimately provide more efficient and targeted breeding strategies for melons [103].

## 3. Molecular Markers, QTLs, and Candidate Genes for Biochemical Compounds in Melon

### 3.1. Sugar Content

Sucrose accumulation during the last phases of fruit development is primarily responsible for the increase in the total sugar content in mature melon fruit. Given that sucrose dominates sugar composition, it is crucial for potential genetic advancements to elucidate the molecular mechanisms underlying sucrose accumulation. Burger et al. [106] discovered that by crossing a low-sucrose cultivar “Faqqous” (*Cucumis melo* subsp. *melo* var. *flexuosus*) with “Noy Yizre’el” (subsp. *melo* var. *reticulatus*), a cultivar with a high sucrose and total sugar content, F_1_ plants had a slightly higher sucrose content than the low-sucrose parent. High sucrose buildup in melon fruit flesh was proposed to be mediated by a single recessive gene based on the segregation of F_2_ and backcross progenies. Diaz et al. [78] constructed a consensus linkage map of melons by merging earlier published QTLs from 18 mapping trials, which included over 10 QTLs related to Brix values, sucrose, and sugars.

Later, Zhang et al. [74] performed the resequencing of “Xinguowei” (sour taste) and “Shouxing” (sweet taste) (parents of the Fengwei melon) cultivars and discovered 1290 polymorphic genes between these 2 parents. Additionally, these authors used SLAF-seq and bulked segregant analysis (super-BSA) to investigate the 2 parents and the F_2_ extreme phenotypes, and discovered 6 sweet trait-related regions containing 13 differential SLAF markers and 62 genes and 23 source-related trait regions with 48 differential SLAF markers and 185 genes. Furthermore, fine-mapping mapped sweet traits to genomic regions on Chr. 6, 10, 11, and 12, and sour traits in the genomic region of Chr. 2, 3, 5, 9, and 12. Subsequently, they located 9 of the 61 differential markers on the parental genome in the sweet and sour trait candidate regions. Of these nine markers, one marker corresponded to sweetness, and eight markers were associated with source-related genes.

The marker, SLAF18745, located in a gene encoding *N-acetylglucosaminyl transferase III* (*MELO3C011944T1*), was correlated with sweetness. Harel-Beja et al. [20] constructed a genetic map and detected 6 significant QTLs for sugar content on Chr. 2, including 668 DNA markers. In addition, one QTL for glucose content in melon fruits was observed on Chr. 4, with the low-sugar parent alleles contributing to a higher glucose content and resulting in the transgressive segregation of this particular trait. Furthermore, several QTLs were found to be related to the sugar content across different locations on Chrs 4, 5, and 7 with one major QTL, *SUCQSC5.1*., on Chr. 5. The authors determined that it was responsible for reducing the soluble solid content (SSC) and sucrose content, and *MELO3C014519* was considered a candidate gene [24].

Plants synthesize sugar in the mesophyll cells of leaves, which is then transported to other plant parts. Shin et al. [107] identified 5173 genes with differential expression, grouping them into 14 clusters based on alterations in their expression patterns. Differentially expressed genes are likely associated with fruit development and maturity via various metabolic pathways. Analysis based on gene set enrichment and pathways revealed that the relevant genes were associated with starch and sucrose metabolism as well as carotenoid biosynthesis during development. Cheng et al. [108] isolated three tonoplast sugar transporters (TSTs), *CmTST1*, *CmTST2*, and *CmTST3*, from melon plants and found that *CmTST 2* played a crucial role in sugar accumulation in melon fruits.

### 3.2. Volatile Aromatic Components

Melons are known for their refreshing aromas, which are attributed to the presence of a mixture of volatile compounds. Esters, aldehydes, alcohols, ketones, and terpenes predominantly contribute to a distinct melon aroma. The profile and concentration of these volatile compounds can vary substantially among different melon varieties, contributing to a wide range of aromatic characteristics from fruity to floral, green, and even herbaceous notes. For instance, in honeydew melon, the principal volatile compound is (E,Z)-2,6-nonadienal, which gives it a fresh, cucumber-like aroma [109]. Cantaloupes are known for the presence of a volatile compound, ethyl-2-methyl butyrate, which imparts a sweet, apple-like fragrance [110]. Additionally, the metabolic pathway responsible for the formation of aroma in melons involves various enzymes, such as lipoxygenases (LOX) and hydroperoxide lyase (HPL) [111].

Zhang et al. [112] have identified 18 *LOX* genes in the melon genome. Their findings, derived from RT-PCR and qPCR expression analyses, indicated varying expression levels across the 18 *CmLOX* genes. These differences were noted in both vegetative and reproductive tissue samples throughout the multiple developmental phases of melon fruit. This suggests that specific *CmLOX* genes play a critical role in producing volatile compounds in oriental melons. Furthermore, Galpaz et al. [73] identified a single significant QTL on Chr. 1 using QTL-seq in the RIL population developed from a cross between PI 414723 and “Dulce.” *CmThAT1* (*MELO3C024190*), encoding S-methyl-thioacetate (thiol acyltransferase), was identified in 10 genes proximal to the QTL genomic region; *S*-methyl-thioacetate, a main constituent of melon fruit aroma, indicated that fruit aroma was controlled by the *CmThAT1* gene.

Mayobre et al. [113] constructed a linkage map using the RIL population developed by “Piel de Sapo” × “Védrantais” and identified 166 QTLs in the melon genome for the aroma profile. Among these, one QTL cluster identified on Chr. 8 was the most crucial contributor to volatile compound biosynthesis, particularly, ethylene-related biosynthesis. Clusters of QTLs linked to esters, lipid-derived volatiles, and apocarotenoids were also detected, suggesting potential genes associated with the biosynthesis of ethyl 3-(methylthio) propanoate and benzaldehyde. These outcomes lay the foundation for more precise mapping projects and substantiate the functionality of the proposed candidate genes, especially those related to aroma generation in melons [113].

Later, Perpina et al. [114] performed GBS on 25 introgression lines, which were developed by integrating specific genomic regions from the Japanese “Ginsen makuwa” (MAK) cultivar into the recurrent ”Charentais” genetic background of the “Védrantais” cultivar. In the study, 57 volatile organic compounds (VOCs) were identified via purge and trap extraction, followed by chromatography–mass spectrometry. The combined analysis of the VOC profiles and GBS genotypes led to the identification of potential genes (such as *MELO3C024886*) encoding *4-coumarate:CoA ligase*, which is potentially a part of the eugenol pathway. The study also highlighted other genes, including *CmLOX18*, *CmAAT*, *CmBAMT*, and *CmCRTISO1*, which have been reported in previous studies as key players in the production of melon VOCs from sources such as fatty acids, amino acids, and carotenoids. Candidate gene and gene-based molecular markers linked with horticultural traits in melons identified in previous studies are reported in Table 2.

## 4. Limitations and Challenges

Although molecular markers facilitate MAB in melons, they are characterized by several limitations and challenges. Marker density in melons is relatively low, which hampers the fine-mapping of crucial agronomic genes. In the realm of crop improvement through molecular techniques, several critical limitations arise. Firstly, there is the intricate challenge of dissecting traits using markers, especially when dealing with gene–environment (G × E) interactions. These interactions often complicate the clarity of results and make the identification of influential genes more arduous. Additionally, a constrained gene pool presents issues, as limited genetic diversity can impede the potential for groundbreaking advancements. From an operational perspective, the financial costs associated with genome analysis and the development of markers and genotyping procedures, particularly in MAS, can be substantial. Even when finances are available, the science itself is not always straightforward; there are inherent limitations in accurately identifying genes and in the subsequent development of gene-based markers. Finally, with the advancements in the field, new traits emerge that await thorough analysis, adding to the list of challenges to be addressed.

Quantitative traits are inherently complex owing to the involvement of multiple genes, making their improvement through molecular markers challenging. Accurate phenotyping is a major obstacle, as environmental factors can cause variations in phenotypic and genotypic traits. Furthermore, short-range linkage disequilibrium complicates marker-trait associations, and functional genomic resources are limited, impeding the precise identification of gene function. Finally, the cost and time-intensive nature of high-throughput genotyping restrict its widespread use, particularly in developing countries. Overcoming these challenges will necessitate concentrated efforts to increase marker density, improve our comprehension of the genetic basis of complex characteristics, enhance phenotyping capabilities, and develop cost-effective genotyping technologies.

## 5. Conclusions and Future Perspectives

In the rapidly evolving field of melon research, genomic tools and markers have provided valuable insights into the intricate genetic underpinnings of various traits. Plant architecture, a fundamental aspect influencing cultivation practices, and sex expression, crucial for reproductive efficiency, have been dissected at the genomic level, enabling the identification of markers that can predict these traits with enhanced precision. Fruit characteristics, including shape, rind color, netting, and flesh color, which substantially influence consumer preferences, have been mapped to specific genomic regions. Notably, markers associated with fruit yield—a critical trait for commercial viability—offer breeders a refined tool to enhance productivity. Seed attributes, encompassing seed size and coat color, have similarly benefited from genomic investigations, with markers elucidating the genetic basis for these variances. Perhaps the most exciting advancements lie in the realm of biochemical traits. Genomic markers linked to sugar content and volatile aromatic components—direct determinants of flavor profiles—are revolutionizing the way we perceive melon quality. These markers provide a roadmap for breeders, enabling them to tailor melon cultivars for optimum sweetness and aromatic appeal.

The past century has witnessed considerable advancements in melon breeding, and we anticipate that this trend will continue. Crop improvement depends largely on conventional breeding approaches. The advent and application of MAS have ushered in a transformative era in melon breeding. By enabling the precise identification and incorporation of desirable genes into breeding lines, MAS has heightened the potential for genetic gains. Traditional breeding often demands extensive time and resources, relying heavily on phenotypic evaluations that may not always translate to the desired genotypic modifications. MAS circumvents this by pinpointing specific genetic markers linked to beneficial traits, allowing breeders to make informed decisions early in the breeding cycle. This precision not only reduces the guesswork inherent in conventional breeding but also substantially expedites the overall breeding process. By accelerating the development of robust melon varieties and enhancing the predictability of desired outcomes, MAS plays a pivotal role in addressing food security challenges and fulfilling the increasing global demand for resilient crops. In essence, through the power of genetic markers and informed breeding strategies, MAS stands as a cornerstone for the future of efficient, effective, and advanced melon cultivation.

Although MAS has increased the effectiveness of crossing and selection, it is limited for crops with complex genetics. The use of DNA microarrays and restriction site-associated DNA (RAD) sequencing will accelerate the genome mapping and tagging of additional QTLs as the cost of genome sequencing decreases. These QTLs may be utilized to introduce resistance into genotypes of high-yielding melons, and several QTLs may be combined with genes with established resistance to other races. A large amount of resequencing data have facilitated the discovery of an increasing number of polymorphic SNPs in melons. The current large-scale sequencing of melon genomes will enable the collection of accurate data on genes with crucial biochemical properties.

Current breeding programs need to incorporate the latest approaches, including whole-genome selection, mutation detection, high-throughput genotyping, genetic transformation, omics, and genome editing, to create critical opportunities to improve fruit quality and accelerate future melon breeding [115]. Genome editing is a potent technique for generating desirable variations using molecular shear and synthetic nucleases. CRISPR/Cas editing can be used to introduce genetic variations into melons in a targeted manner, which can accelerate crop improvement. Further research is necessary to identify new resources for genes associated with specific climate change-induced issues, and special quality traits of melon fruit [116]. Additional studies are required to identify novel genes involved in pathogen resistance, development, ripening, and other attributes related to the quality of melon fruits. These genes can be identified from already available wild germplasms by comprehensive analysis of genomics, transcriptomics, metabonomics, and bioinformatics [116]. With the advent of advanced genomics, markers associated with these traits are increasingly being identified, providing an avenue for precision breeding. The genomic understanding of these traits offers a promising future for the development of melon varieties that cater to specific market needs and consumer preferences, combining the best of agronomic and sensory properties.

## Figures and Tables

**Table 1:** Genetic linkage maps and molecular markers for the quantitative trait loci (QTLs) of horticultural traits in melons.

Traits	QTL Name	Parents	Generation ^a^	Linkage Group	Markers	Reference
Fruit shape	*fs1.1*	USDA-846-1 × Top Mark	RIL	I	OPAL11-1250	[19]
	*fs1.2*			I	OPP12-564	
	*fs2.3*			II	E14M50-159	
	*fs6.4*			VI	OPR5-500	
	*fs7.5*			VII	OPAD15-830	
	*fs11.6*			XI	OPAO7-600	
	*fs11.7*			XI	TJ23	
	*fslg9.8*			IX	E25M17-165	
	*fsqs2.1*	Piel de Sapo × PI124112	F_2_	II	CMPSNP431-AIuICAPS	[12]
	*fsqs8.1*			VIII	GCM241-PSI_25-H03	
	*fsqs12.1*			XII	AI_35-A08-CMPSNP361	
	*fsh8.1*	PI 414723-S_5_ × Dulce	RIL	VIII	NA	[20]
	*fsh2.1*			II	NA	
Fruit length	*flqs2.1*	Piel de Sapo × PI124112	F_2_	II	CMPSNP431-ECM61	[12]
	*flqs3b.1*			IIIb	ECM205-CMPSNP998	
	*flqs6a.1*			VIa	ECM52-CMTCN41	
	*flqs8.1*			VIII	GCM241-PSI_25-H03	
	*fl2.1*	PI414723-S_5_ × Dulce	RIL	II	NA	[20]
	*fl8.1*			VIII	NA	
	*FL1*	PI435288 × C940-fe	F_2_	I	CMGAN271-DE1240	[21]
	*FL2*			II	DE1468-CMN038	
Fruit weight	*fw3.1*	Piel de Sapo × PI161375	DHL	III	CSAT425B-CM139	[22]
	*fw4.1*			IV	CMAT35-MC256	
	*fw5.1*			V	CMTC160-MC264	
	*fw12.1*			XII	MC226-MC8	
Soluble solid content	*ssc1.1*	USDA-846-1 × Top Mark	RIL	I	TJ27	[19]
	*ssc2.2*			II	OPAD14-400	
	*ssc6.3*			VI	OPAI8-800	
	*ssc7.4*			VII	E19M51-302	
	*ssc7.4*			VII	E24M48-133	
	*ssc8.5*			VIII	OPAY1-831	
	*ssc8.6*			VIII	OPAY16-400	
	*ssc9.7*			IX	CMATN22	
	*ssc10.8*			X	CMGA172	
Flesh color	*flc2.1*	PI414723-S_5_ × Dulce	RIL	II	NA	[20]
	*flc6.1*			VI	NA	
	*flc8.1*			VIII	NA	
	*FC1*	PI435288 × C940-fe	F_2_	I	CMN238-DM0085	[21]
	*FC2*			II	CMN04_19-CMMS35_5	
Percent netting at full slip	*pn2.1*	USDA-846-1 × Top Mark	RIL	II	OPAI9-250	[19]
	*pn5.2*			V	CMTCN9	
	*pn6.3*			VI	OPO6-1375	
	*pn8.4*			VIII	OPAH14-831	
	*pn11.5*			XI	CMGA104	
Net density	*ntd2.1*	PI414723-S_5_ × Dulce	RIL	II	NA	[20]
	*ntd 2.2*			II	NA	
	*ntd 2.3*			II	NA	
	*ntd 2.4*			II	NA	
	*ND*	PI435288 × C940-fe	F_2_	NA	TJ24-SSR02042	[21]
Total carotenoid	*car6.1*	PI 414723-S_5_ × Dulce	RIL	VI	NA	[20]
	*car8.1*			VIII	NA	
β-carotene	*_β_cr6.1*	PI414723-S_5_ × Dulce	RIL	VI	NA	[20]
	*_β_cr2.1*			II	NA	
	*_β_carM.8.1*	Chinese melon line ‘Q 3-2-2′ × Top Mark	F_3_	VIII	CMN21_25	[23]
	*_β_carM.91*			IX	Or	
	*_β_carE.6.1*			VI	CMTCN41	
	*_β_carE.9.1*			IX	Or	
Sugar content	*SUCQSC5.1*	Piel de Sapo × Songwhan Charmi	NIL	V	CMPSNP437	[24]
Primary branch	*Pb1.1*	USDA 846-1 × Top Mark	RIL	I	OPAE3-600	[25]
	*pb1.2*			I	CMGAN25	
	*pb2.3*			II	E13M51-284	
	*pb8.4*			VIII	OPAL8-400	
	*pb10.5*			X	OPAB4-750	
	*pb12.6*			XII	CMCTN1	

^a^ recombinant inbred line (RIL), near isogenic line (NIL), double-haploid line (DHL), not available (NA).

**Table 2:** Candidate genes and gene-based molecular markers linked with horticultural traits in melon.

Traits	Locus Name	Chr.	Gene ID	Gene Function	Marker Name (Type ^a^)	Primer Sequence (5′-3′)	References
Sex expression	*A*	NA	*CmACS-7*	1-aminocyclopropane-1-carboxylic acid synthase (ACS)	T1ex (SCAR) EX1_C170T (CAPS)	F: AACGGATGAAGAAGGAAAACGAAG R: ATATTGGGCAGTGTCCACACAAAA F: TTGGCTCTCAAAAAGGGAAA R: CCCTCACAATTTTCCTCCAA	[49]
Male sterility	NA	9	*MELO3C021653T1*	Transcription factor ABORTED MICROSPORES isoform X1	AMS	F: CGCTGGGACTGAGAACAATA R: TAGCCAGTTGGGTTCATTTG	[72]
Fruit rind color	NA	4	*(CmMt1*) *CmAPRR2* *MELO3C003375*	Two-component response regulator-like protein APRR2	(CmAPRR2^SNP(G/T)^) (KASP)	F: GTTTATTAGGTGGACTGGACCCCAG R: GTTTATTAGGTGGACTGGACCCCAT	[15,64]
	NA	2	(*CmMt2*) *MELO3C026282*	Sec14p-like phosphatidylinositol transfer family protein	CmSNP19 (KASP)	F:TCAGCGGCCTCAAATGAAG R: TCAGCGGCCTCAAATGAAA	[15]
	NA	8	*MELO3C003097*	SLOW GREEN 1	NA	NA	[64]
Fruit shape	*CmFSI8/CmOFP13*	8	*MELO3C025206*	Transcription repressor, Ovate Family Protein 1 (OFP1)	ID-FS6 (CAPS)	F: CTCGCCCCCACAGTTCTAAA R: TGATAATGTCACACACACGCA	[16]
					ID-FS8	F: ATGGGGACAAACTCTGAAGACC R: GACGAAGTAGGCATCGTTGGA	
	NA	6	*MELO3C006884*	Protein IQ-DOMAIN 14-like	CmSUN23-24	F: ATTTGACAACTCGGCACTTCTG R: TCTAACCAACTTGACCCGACTG	[71]
	NA	4	*MELO3C013004*	Protein IQ-DOMAIN 14-like	CmSUN25-26-27c	F: TTTCTTCTCCACTCCCTTGTCG R: ATCGTGGAGTGCTCTGTGCC	[71]
Flesh color	*gf* orange/non orange	9	(*CmOr*) *MELO3C005449*	Protein ORANGE-ORANGE, chloroplastic	OR CAPS	F: CTCCTTGGTTTTCTTCATG R: CGACTTCGAATGTTCTCC	[67]
	*Wf*	8	*MELO3C003097*	SLOW GREEN 1	NA	NA	[64]
		8	(*CmPPR1*) *MELO3C003069*	Pentatricopeptide repeat-containing family protein	CAPS	F: CTACCTCCGCTTCCATTG R: TCGTCACAAAGTCCCAAAG	[73]
Sweetness	NA	10	*MELO3C011944T1*	N-acetylglucosaminyl transferase III	SLAF18745	NA	[74]
Seed coat color	*CmBS-1*	6	*MELO3C019554*	Homeobox protein	NA	NA	[75]
Biochemical compound (Thiol acyltransferase)	NA	1	(*CmThAT1*) *MELO3C024190*	Acetyl-CoA acetyltransferase	NA	NA	[73]

^a^ sequence-characterized amplified region (SCAR), cleaved amplified polymorphic sequences (CAPS), Kompetitive allele-specific PCR (KASP), specific length amplified fragment sequencing (SLAF), not available (NA).

## Data Availability

Data are contained within the review.
